# Development of Network Security Based on the Neural Network PSD Algorithm

**DOI:** 10.1155/2022/9460985

**Published:** 2022-09-30

**Authors:** Jianxun Li, Song Ji, Yiran Jiang

**Affiliations:** School of Information Science and Engineering, Baoding University of Technology, Baoding 071000, Hebei, China

## Abstract

The more frequent occurrence of network security incidents has an impact on network security. Through the research on network security situational awareness, this paper constructs a multilevel network security situation evaluation index system from various aspects and uses the Elman neural network optimized by the genetic algorithm to evaluate network security situation. Aiming at the disadvantage of subjective dependence in the traditional assignment method of basic probability assignment function, Elman neural network is used to obtain the basic probability assignment function to increase its objectivity, and it is optimized with the PSD algorithm. In addition, the neural network is further improved by the genetic algorithm. In the traditional D-S evidence theory, an evidence correction step is added to optimize the situation that the final judgment result is incorrect due to evidence conflict. Finally, the fusion rules of the D-S evidence theory are used to fuse the support degrees of the four first-level situations to different security levels to obtain the final network security situation assessment result. The results show that the prediction accuracy of the GA-Elman neural network model is as high as 80%, which is significantly higher than that of the traditional D-S model, indicating that the model proposed in this paper has improved the accuracy of the assessment and prediction results. In conclusion, this study provides feasible theoretical prediction guidance for the accurate assessment of network security posture, reveals the improvement ideas for network security development, and is of great significance for the maintenance of network environment security.

## 1. Introduction

The rapidly developing economy has provided development opportunities for the field, and network-related technologies have been widely used [1, 2]. In the long-term social development process, people find that the rational use of the Internet can improve the way of life and make people's activities more convenient. However, with the continuous expansion of the network application scope and the continuous increase in the frequency of use, network security incidents occur more and more frequently, which has an impact on network security. Due to the expansion of network usage, network security incidents occur frequently. The popularization of the network provides convenience to people's lives, but it also brings greater difficulty to network security maintenance [3, 4].

Network attack is a typical representative of network security incidents [5], and data leakage is one of the serious consequences of network attacks. The total number of data breaches in 2020 surpassed the previous maximum and reached a new high, becoming the highest number of breaches so far. The frequent occurrence of security incidents shows the importance of paying attention to the security of the network environment [6]. In 2014, the state made it clear that in order to ensure national security, the maintenance of network security as a part of it is also crucial. However, because the defense side of traditional network security defense technology does not really combine data, intelligence, and other information resources with network security, some single security technology is relatively backward, and it is necessary to achieve a high level of network security protection. It is not enough to use only one-sided technologies such as firewall, intrusion detection, and access control [7]. The birth of network security situational awareness technology has solved the problems existing in traditional security technology. The emergence of this technology enables the protection terminal to take the relevant protection measures proactively while detecting whether a network security attack occurs or not and performs real-time feedback and operations [8, 9]. Compared with foreign countries, China's network security situational awareness research started relatively late and has gone through many stages. As China's research on SOC/SIEM and other types of products and technologies has been going on for many years, it has always been at the level of alerting security incidents and has not provided any guarantee for protecting network security. The emergence of big data technology has made a breakthrough for domestic researchers and realized the security analysis technology based on big data [10]. Through the research on network security situational awareness, academic personnel at home and abroad have achieved many remarkable results. In order to improve the accuracy of network security situation assessment, Siaterlis et al. [11] proposed a new network security situation assessment scheme by combining evidence theory with situation assessment. Dutt et al. [12] proposed an IBLT model based on tools to resist security attacks, which can identify some behaviors in the network environment, thereby effectively improving the accuracy of evaluation. Almeida et al. [13] connected modular components in a multilayered manner, proposing a situational awareness model based on EXEHDA-ISSA. Zhang and He [14] used big data analysis and combined forecasting methods to predict the security situation of smart grids; Liu [15] improved the ability of D-S evidence theory to confront conflict by proposing predictive models based on improved D-S.

Network security situational awareness is one of the hot research points in the current network security direction. The reason is that it can dynamically show the overall situation of network security by evaluating the security influencing factors at various levels and, at the same time, makes accurate predictions on the development trend of future network security situation [16]. This paper selects the method of combining spiking neural network (PSD) to evaluate and predict the network security situation. The optimized neural network assigns BPA to each feature, uses the PSD algorithm to process the input of relevant data, improves the parameter optimization ability of the system, and obtains more accurate results while improving the objectivity of BPA assignment. At the same time, this paper corrects the BPA by introducing the Pearson coefficient and the distance of evidence to solve the problem of wrong conclusions caused by conflicting evidence.

## 2. Establishment of the Theoretical Model

### 2.1. DS-GAElman Evidence Theory

#### 2.1.1. D-S Evidence Theory Synthesis Rules

The specific formula of the synthesis rule of D-S evidence theory [17] is as follows:

(1) Two mass function synthesis rules are
  (1) $$\begin{array}{c} m=m_{1}⊕m_{2},\left\{\begin{array}{c} m\left(⊘\right)=0\text{,}\\ m\left(A\right)=k^{-1}, \end{array}\right.\underset{A_{1}\cap A_{2}=A}{\sum }m_{1}\left(A_{1}\right)\cdot m_{2}\left(A_{2}\right)\text{,} \end{array}$$
where $k=1-\sum_{A_{1}\cap A_{2}+\emptyset }m_{1}\left(A_{1}\right)\cdot m_{2}\left(A_{2}\right)=\underset{A_{1}\cap A_{2}+\emptyset }{\sum }m_{1}\left(A_{1}\right)\cdot m_{2}\left(A_{2}\right)$.
(2) Multiple mass function synthesis rules are

(2) $$\begin{array}{c} m=m_{1}⊕m_{2}⊕\ldots m_{n}\left\{\begin{array}{c} m\left(⊘\right)=0\text{,}\\ m\left(A\right)=k^{-1}, \end{array}\right.\underset{A_{1}\cap A_{2}\cap \cdots A_{n}=A}{\sum }m_{1}\left(A_{1}\right)\cdot m_{2}\left(A_{2}\right)\cdot \ldots \cdot m_{n}\left(A_{n}\right)\text{,} \end{array}$$

where $k=1-\underset{A_{1}\cap A_{2}\cap \ldots A_{n}=\emptyset }{\sum }m_{1}\left(A_{1}\right)\cdot m_{2}\left(A_{2}\right)\cdot \ldots \cdot m_{n}\left(A_{n}\right)=\underset{A_{1}\cap A_{2}\cap \ldots A_{n}=\emptyset }{\sum }m_{1}\left(A_{1}\right)\cdot m_{2}\left(A_{2}\right)\cdot \ldots \cdot m_{n}\left(A_{n}\right)$.

#### 2.1.2. Improvement of D-S Evidence Theory

After a lot of research, researchers believe that D-S evidence theory can be selected as a solution [18] because the theory can describe uncertainty and has the ability to solve such problems. However, in the process of using evidence theory, researchers have found that there may be problems such as contradicting the actual results, unfairness, or even being rejected by one vote when fusing evidence. Among these emerging problems, the fusion results of highly conflicting evidences that contradict intuitive results have become the focus of researchers. When the D-S evidence theory fuses evidences with less conflict, the error between the final synthesis result and the correct result will be smaller, and when there is high conflict between the evidences, the synthesis result of the D-S evidence theory will have a larger deviation even against the correct result. In this regard, this paper calculates the credibility of evidence by introducing Pearson correlation and calculates the average evidence probability according to the credibility [19]. The evidence weight *w* is determined, and *w* is used as a correction coefficient to modify the weight of the original evidence body, so as to obtain the improved evidence BPA. Finally, all BPAs are synthesized using the basic synthesis rules to obtain the final result. The improved steps are as follows:

(1) Calculate the similarity of the Pearson correlation coefficient between each evidence:*x* and *y* are two variables, and assuming that there is a certain linear relationship between *x* and *y*, this correlation can be represented by the Pearson correlation coefficient *P* ranging from −1 to 1. When P is a number greater than 0, it means that any one of the variables in *x* and *y* increases with the increase of the other variable. This situation indicates that the relationship between *x* and *y* is a positive correlation. Conversely, when *P* is negative, it indicates that the relationship between *x* and *y* is linearly negative. When one variable in *x* and *y* increases, the other value decreases instead. If *P* is equal to 0, it means that there is no linear correlation between *x* and *y*. The formula for the correlation coefficient is as follows:
  (3) $$\begin{array}{c} P_{ij}=\frac{\text{cov}\left(m_{i},m_{j}\right)}{\sigma_{m_{i}}\sigma_{m_{j}}}=\frac{E\left(\left(m_{i}-\mu_{m_{i}}\right)\left(m_{j}-\mu_{m_{j}}\right)\right)}{\sqrt{E\left(m_{i}^{2}\right)-E^{2}\left(m_{i}\right)}\sqrt{E\left(m_{j}^{2}\right)-E^{2}\left(m_{j}\right)}}\text{.} \end{array}$$
In the formula, *μ*_*m*_*j*__=*m*_*i*_ − *E*(*m*_*i*_), *μ*_*m*_*j*__=*m*_*j*_ − *E*(*m*_*j*_). The correlation coefficient of the above two evidences can be extended to *n* × *n* evidences, and the correlation matrix *P*_*ij*_ can be obtained as follows:
  (4) $$\begin{array}{c} P_{ij}=\left[\begin{array}{ccccc} p_{11} & p_{12} & \cdot & \cdot & p_{1n}\\ p_{21} & p_{22} & \cdot & \cdot & p_{2n}\\ \cdot & \cdot & \cdot \\ \cdot & \cdot & \cdot \\ \cdot & \cdot & \cdot \\ p_{n1} & p_{n2} & \cdot & \cdot & p_{nn} \end{array}\right]\text{.} \end{array}$$
(2) Calculate the support degree of evidence *m*_*i*_:
  (5) $$\begin{array}{c} \sup_{m_{i}}\;=\overset{n}{\underset{j=1,j\neq i}{\sum }}P_{ij}\left(m_{i},m_{j}\right)\text{.} \end{array}$$
(3) Calculate the credibility of the evidence:By analyzing the support formula of *m*_*i*_, it can be seen that the greater the correlation between the two evidences, the higher the support, then the higher the credibility of *m*_*i*_, so the credibility of *m*_*i*_:
  (6) $$\begin{array}{c} {de\;p}_{m_{i}}=\frac{{sup}_{m_{i}}}{\overset{n}{\underset{i=1}{\sum }}{sup}_{m_{i}}},{de\;p}_{m_{i}}\in \left[0,1\right]\text{.} \end{array}$$
(4) Calculate the adaptive weight *w*_*i*_ separately for different hypothesis results:The smaller the *d*_*i*_, the closer the distance to the average evidence probability, the greater the weight of the evidence. It can be known that if the distance is inversely proportional to the weight, then the probability weight *w*_*i*_ of each evidence is given as
  (7) $$\begin{array}{c} w_{i}=\frac{1/d_{i}}{\overset{n}{\underset{i=1}{\sum }}1/d_{i}}\text{.} \end{array}$$
(5) Calculate the probability *m*_*i*_′ of each evidence separately:
  (8) $$\begin{array}{c} m_{i}=w_{i}m_{i}\text{.} \end{array}$$
(6) All *m*_*i*_ are fused sequentially using the synthesis rules, and after all the iterative syntheses are completed, the situation that meets the decision rules is selected as the final result of the problem.

### 2.2. Neural Network Model and Its Improvement

Feedforward neural networks are only affected by the input data at the current moment and do not care about the data at the previous moment [20, 21]. However, when solving practical problems, it will be found that there are intricate relationships among many data, including certain time series. If only the data at the current moment is considered, the final output may be biased, and the feedforward neural network does not have the ability to remember and cannot effectively deal with timing problems. Therefore, recurrent neural networks have been considered to solve this type of problem [22].

As the basic element of information transmission, Elman neural network was first proposed by Elman [23]. It adds an additional layer to the hidden layer to record the historical data of the hidden layer, that is, the context layer. Because of its dynamic and nonlinear characteristics, it is very sensitive to time series data, and the data have complex correlation. Elman neural network is selected as the core module to obtain BPA, and the PSD learning algorithm is used for deep learning and optimization [24].

#### 2.2.1. Elman Neural Network Model

Compared with the ordinary neural network structure, the Elman network adds a new layer of undertaking layer, the implicit layer transmits the processed data to the undertaker layer, the undertaker layer memorizes the information transmitted by the implicit layer and combines the received data with the input layer of the next moment as the input of the implicit layer at the next moment [25, 26]. By storing it through the undertaker layer and outputting it to the hidden layer at the next moment, the neural network has the function of dynamic memory recognition of historical input data and enhances its ability to process dynamic information. The specific mathematical model is given as

(9) $$\begin{array}{c} \left\{\begin{array}{c} h\left(k\right)=g\left(w_{3}\cdot q\left(k\right)\right)\text{,}\\ q\left(k\right)=f\left[w_{1}\cdot q_{c}\left(k\right)+w_{2}\left(u\cdot \left(k-1\right)\right)\right]\text{,}\\ q_{c}\left(k\right)=q\left(k-1\right)\text{,} \end{array}\right. \end{array}$$

where *h* is the output of the output layer, *g*()is the transfer function of the output layer, *w*_3_ is the weight of the data processed by the implicit layer in the data received by the output layer, *q* is the state of the implicit layer, and *k* is the current moment. In the second formula, *f* is the handling function of the implicit layer, and in most cases, Sigmoid is selected, and *w*_1_ refers to the weight of the data processed by the implicit layer in all the received data of the undertaken layer, *w*_2_ is the weight of the information received by the input layer to the implicit layer, and *u* is the input of the input layer. *q*_*c*_ in the second and third formulas refers to the state that is output for the undertaker layer, and *k* − 1 in *q* indicates the previous moment.

#### 2.2.2. Improvements to Elman Neural Networks

The traditional Elman neural network takes the gradient descent method as its weight update method, which is easy to fall into the local minimum value in the case of multiple local minimum values in the error function; in this case, this method cannot guarantee that the optimal solution to be solved can be found, which will have a bad impact on the network training effect, so that the error of the final output result increases [27].

(1) Initialize the Elman neural network structure:Set related parameters including the number of neurons in each layer of the Elman neural network.
(2) Genetic algorithm parameter initialization:Set relevant parameters such as population size, probability of crossover and mutation, and target scalar function.
(3) Code to generate the initial population:The weights and thresholds of the Elman neural network are used as parameters to be optimized, and they are encoded as an individual, and multiple individuals form a population.
(4) Determination of fitness function:The genetic algorithm needs to assign the individual probability through a certain index and provides the basis for the genetic algorithm to survive the fittest through the size of the selected probability. The fitness function is used as an index to evaluate the pros and cons of chromosomes, and it needs to use a specific function as an evaluation criterion. Therefore, the fitness function needs to be used to calculate the fitness value of each individual, and the individual in the group can be distinguished from the bad by comparing the size of the function value. The fitness function *f* in this paper is
  (10) $$\begin{array}{c} f=\frac{1}{\overset{n}{\underset{i=1}{\sum }}y_{i}^{\prime }-y_{i}}\text{.} \end{array}$$
In the formula, *f* is the fitness value, *y*_*i*_′ and *y*_*i*_ are the actual and expected weights and thresholds output by the *i*th output node, and *n* is the total number of network input samples.
(5) Perform the selection operation:According to the fitness function set in (d), it can be seen that the greater the fitness, the better the individual, and the better the genes of the individual in the next generation. Therefore, the probability of the individual being selected can be improved by increasing the selection probability. In this paper, the roulette method is used as the method of selection operation, and the probability of each individual being selected is
  (11) $$\begin{array}{c} P_{i}=\frac{f_{i}}{\overset{n}{\underset{i=1}{\sum }}f_{i}},1\leq i\leq n\text{.} \end{array}$$
The formula is to take the ratio of the individual fitness value to the sum of all individual fitness as the probability of the individual being selected.
(6) Obtain the optimization result: Determine whether the obtained results meet the training requirements and whether the termination conditions are met. If it is reached, pass the obtained optimal initial weights and thresholds to the Elman neural network, ending the iterative process. If the termination condition is not reached, the iterative evolution is continued until the condition is finally reached, then the iteration ends, and the result is obtained.

#### 2.2.3. PSD Learning Algorithm

By analogy with the Widrow–Hoff (WH) learning rule, we can get

(12) $$\begin{array}{c} \Delta w_{i}=\alpha x_{i}\left(y_{d}-y_{o}\right)\text{,} \end{array}$$

where *w*_*i*_ is the weight of the corresponding quantity of the *i*th input, *α* is the learning rate, *y*_*d*_ is the expected sequence, *y*_*o*_ is the actual output sequence, and *x*_*i*_ is the input sequence. Because the output is actually a sequence containing pulse spikes, it is very difficult to obtain the derivation. In the PSD rule, a sharp pulse and a convolution kernel are convolved to obtain a derivable continuous value:

(13) $$\begin{array}{c} K\left(t-t^{j}\right)=V_{0}\cdot \left(\exp\left(\frac{-\left(t-t^{j}\right)}{\tau_{s}}\right)-\exp\left(\frac{-\left(t-t^{j}\right)}{\tau_{f}}\right)\right)\text{.} \end{array}$$

## 3. Cybersecurity Situation Assessment

The selection of security situation indicators is the basis of the multilevel index system, and it is also the basis for the data sources and situation level standards of the security situational awareness process in the following sections. The results of the selection of security situation indicators directly affect the accuracy and rationality of subsequent security situation assessment and security situation prediction results. Because the network security environment is complex and diverse, certain selection rules need to be adopted when processing the data indicators of the network security situation. In general, the major functional modules of the network security system should be covered [28]. However, if too many indicators are selected, it is difficult to determine the importance and weight of each indicator. It will also increase the overall complexity of the situational awareness model and affect the situation assessment and prediction results. Therefore, it is necessary to obtain more comprehensive and accurate security situation indicators according to reasonable principles.

### 3.1. Construction of the Indicator System

According to the principle of hierarchy and classification, this paper proposes a relatively complete model of the first situational awareness index system, and according to the classification, the indicators are divided into four indicators: vulnerability, disaster tolerance, threat, and stability. The function of the first step is to select several main indicators, and these indicators need to have high independence and strong generality and use them as first-level indicators. Then, for each first-level indicator, a certain number of second-level indicators are selected as auxiliary indicators to describe the security situation. The network security situation assessment index system constructed is shown in Table 1.

### 3.2. Quantification of Indicators

As can be seen from the description in the previous section, there are many kinds of factors that affect the security of the network environment, and the raw data formats of these factors are different. In addition to differences in format, there are many other situations that make these data unsuitable for direct experimental manipulation since the vast majority of manipulations use numeric data. Therefore, we need to quantify the collected data on the basis of CVSS. Some of the formulas are mentioned in the next section.

#### 3.2.1. Degree of Network Vulnerability

In the vast majority of security incidents, vulnerabilities in the network environment are the main reason for cyber-attacks. Vulnerabilities vary in severity and impact on the network. The quantitative formula for the degree of network vulnerability is mainly composed of the number and category of vulnerabilities. The formula is as follows:

(14) $$\begin{array}{c} b=\frac{\sum_{k=1}^{n}\sum_{t=1}^{s}w_{k1}I_{k}N_{kl}}{B_{n}}\text{.} \end{array}$$

Among them, b represents the vulnerability level, *B*_n_ represents the total number of network vulnerabilities, *n* represents the number of devices, *S* represents how many different vulnerabilities exist, *k* represents the number of devices, *I* represents the number of vulnerabilities, and *w*_*k*1_ represents the weight of vulnerabilities, and this value is given a specific score by the CVSS standard, and *N*_*kl*_is the number of certain vulnerabilities contained in a certain device.

#### 3.2.2. Network Topology

When in a real network environment, the network topology will also have a certain impact on network security [29]. Since topology is used to determine how devices are placed, their security and degree of security are also key objectives to consider when measuring network security. The formula used is as follows:

(15) $$\begin{array}{c} \text{topo}=\overset{n}{\underset{k=1}{\sum }}TP_{k}\text{,}\\ TP_{i}=\left\{\begin{array}{c} 1.0,n\in \left[0,4\right]\text{,}\\ 0.6,n\in \left(4,6\right]\text{,}\\ 0.2,n\in \left(6,+\infty \right)\text{,} \end{array}\right. \end{array}$$

where topo is the network topology quantization value, and *TP*_*k*_ refers to the number of nodes present in the environment measured by *n* in the fractional metric of the topology used by a network.

#### 3.2.3. The Severity of the Attack

The destruction of the network environment after being attacked is a factor affecting network security. The broken situation after being attacked generally increases. At the same time, it is also necessary to consider that the degree of danger of different devices under the same attack is different, and the degree of damage to network security caused by different types of attacks is also different. Therefore, the quantification formula of attack severity is as follows:

(16) $$\begin{array}{c} A=\frac{\sum_{k=1}^{n}\sum_{i=1}^{s}r_{k}b_{k}Q_{kl}}{a_{n}}\text{.} \end{array}$$

In the formula, *n* is the total number of devices, *S* is the number of types of attacks that are suffered, *r*_*k*_ refers to the level of a host receiving a certain attack, which is derived from the degree of possible impact of the attack, *Q*_*kl*_ refers to how many times the host has been attacked of a certain type, *b*_*k*_ refers to the degree of impact of a vulnerability, *a*_*n*_ represents the total number of types of attacks in a certain period of time, and *A* is a score that refers to the degree of damage caused by the attack.

#### 3.2.4. The Degree of Occurrence of Attacks

The number of security incidents in a certain period of time will also affect the score of the entire situation indicator. If the number of security incidents occurs in a certain period of time, it means that the security of the environment is low. On the contrary, if the number of security incidents is small, the network security is relatively high. Therefore, the quantitative formula for the frequency of attack incidents is

(17) $$\begin{array}{c} f=\frac{N}{t}\text{,} \end{array}$$

where *f* represents the occurrence of attack events, *t* is a fixed time period, and *N* represents the total number of attack events in the time period *t*.

### 3.3. Criteria for Classification of Safety Levels

The network situation is divided into five security levels, as shown in Table 2. Class I indicate that the current network security is high. At this time, the status of various indicators of the network has reached an excellent level, and it is in a state of continuous security and is under attack. Class II indicates that the network is currently in a relatively secure state, and the security posture of the network is in a good state, but compared to class I, the security state has been slightly affected. Class III means that the network situation is basically safe, and the network is in a medium security state. The network may have been attacked. Although the situation is basically safe, it is necessary to focus on some indicators in the poor state to prevent the network security state from deteriorating. Class IV indicates that the network is under moderate threat and may have suffered a certain degree of attack. Security personnel need to immediately look for indicators that are under moderate threat, and immediately take reasonable measures to make the network security state out of the threat state and restore security. Class V indicates that the system is extremely dangerous at this time, and the network security has been seriously affected. Emergency measures must be taken to eliminate the danger immediately.

Before the evaluation, it is necessary to process the data collected in the real network environment that may have some duplicate information and incomplete information. The processed dataset is divided based on a sliding window method, and a sample set is generated , some of which are used for neural network training, and the remaining part of the data is used as a test set for evaluation and testing, and the trained GA-Elman neural network is used to evaluate the model. BPA acquisition of indicator evidence is required to carry out the D-S evidence theory. The weight value of each evidence is revised, and then the updated basic probability distribution function is obtained, and finally, the evidence is iteratively fused through fusion rules. The final output value of the GA-Elman neural network model is the situational BPA. Therefore, this paper establishes the corresponding relationship between the situational level and the situational BPA, as shown in Table 3. The maximum confidence of the BPA is taken as the result, and the final decision of the network security posture assessment result is made according to the assessment level table and the correspondence table, and the assessment result is output.

### 3.4. Data Analysis of Network Security Situation Based on DS-GA-Elman

The experimental data in this paper are the public dataset NSL-KDD [30]. Most of the public datasets are real, and effective data are obtained by a well-known laboratory which had been collecting network traffic data for several months, and the dataset has been used by many researchers. Therefore, the comparison of experimental results using this dataset has certain comparability [31].

After the processing of the GA-Elman evaluation model, this paper obtains the support of each sample for different network security levels. Then uses the improved D-S evidence theory to fuse the BPA after correction and finally takes the fusion result with the largest value as the final evaluation result. According to the corresponding relationship between the situation level and situational BPA established in Table 3, the security level is evaluated. Situation states [O1, O2, O3, U] represent A-level security state, B-level security state, dangerous state, and unknown state, respectively. A-level security state only includes class I, and B-level security includes class II and III security levels. Hazardous states include class IV and class V safety levels. The final BPA value is shown in Table 4.

Then, the final BPA results obtained by each sample are classified according to the network security classification standard, and the following conclusions are obtained (Table 5).

**Figure 1:**
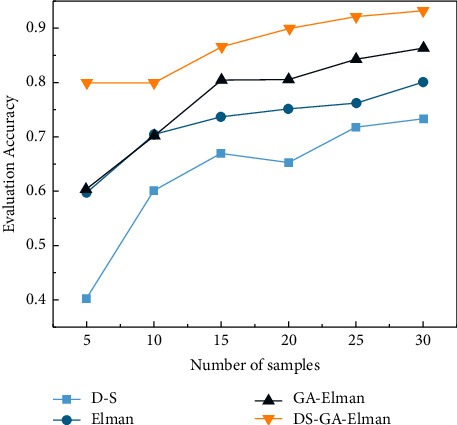
Comparison of accuracy of each model.

From Table 6, the error rate of using the traditional D-S evidence theory to evaluate the network security situation is as high as 30%. Compared with the D-S evidence theory, the number of errors in the situation assessment results of the Elman neural network increased by 10%, and the correct rate was only 60%. The results based on DS-GA-Elman neural network has an accuracy rate of 80% for the evaluation results of the test set, which is 10% higher than that of the traditional D-S evidence theory situation assessment results. The evaluation results of the Elman neural network model have a high accuracy rate. In order to verify the accuracy of the model evaluation, different samples and the number of samples were randomly selected many times for experiments. The comparison of the evaluation accuracy of each model is shown in Figure 1.

### 3.5. DEIPSO-GRU Prediction Model Results and Analysis

**Figure 2:**
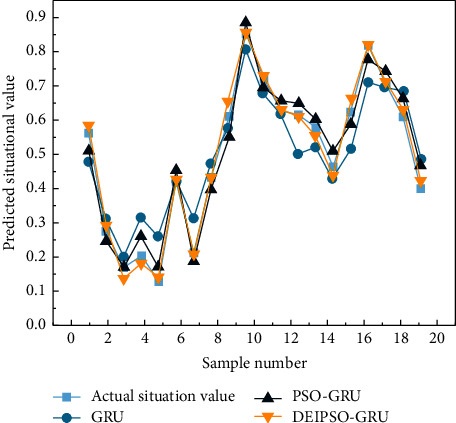
Multimodel prediction of situational value.

This paper compares the prediction results of the basic GRU and PSO-GRU-based prediction methods with the situation prediction results of the DEIPSO-GRU-based prediction method. The prediction results of multiple models are shown in Figure 2. It can be seen that the four forecasting methods show roughly the same trend of change. The maximum predicted value was achieved in sample 9.

**Figure 3:**
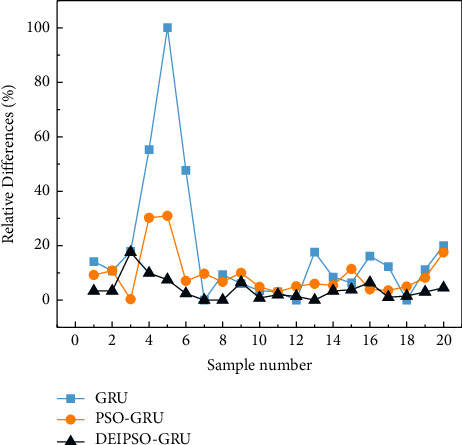
Relative error comparison between GRU, PSO-GRU, and DEIPSO-GRU.

For predicting the prediction results of each model, the relative error formula *RE*=|*y*_*i*_ − *y*_*i*_′|/*y*_*i*_′ × 100% and the average absolute percentage error $\text{MAPE}=\sqrt{\sum_{i=1}^{n}{\left(\left(y_{i}^{\prime }-y_{i}\right)/y_{i}^{\prime }\right)}^{2}}/n$ are used for error comparison and analysis, where *y*_*i*_′ represents the true situation value, *y*_*i*_ represents the output value of the model, *n* is the number of prediction samples, and the lesser the MAPE is, the more accurate the prediction results are. The average percentage error values are shown in Table 7. The *RE* value comparison is shown in Figure 3.

As can be seen from the prediction results in Figure 3, the GRU showed higher predictive values than the other two prediction models. In particular, at sample 5, it reaches a maximum of 102. The DEIPSO-GRU security situation prediction model is basically consistent with the trend of the real situation. Only a few predicted values differ from the actual situation values, and most of the predicted situation values and the situation change curves are consistent with the actual situation value change trends. However, some of the prediction results of the basic GRU prediction model and the traditional PSO optimized GRU prediction model has a large deviation from the actual situation value. From the figure, there is a large error with the actual situation value. At the same time, in Figure 3, the error between the prediction results of the DEIPSO-GRU model and the actual situation value is relatively small, and it is relatively lower than the other two prediction models in most cases. Compared with the traditional GRU structure prediction, the accuracy of the GRU prediction model optimized by the PSO algorithm has been improved, but there are still many prediction deviations. The predicted MAPE values of this model are relatively low, but still fall short. However, the prediction model based on DEIPSO-GRU neural network has a relatively stable performance and the lowest MAPE value when making predictions. Therefore, the network security situation prediction model based on the DEIPSO-GRU structure is feasible and effective, and the prediction accuracy rate is improved.

## 4. Conclusion

This paper proposes a network security situation assessment model based on DS-GA-Elman neural network. The advantage of neural network is used to obtain the basic probability distribution function required in the synthesis rule of D-S evidence theory, and the disadvantage of the subjective dependence of traditional BPA is removed. The specific conclusions are as follows:

1. The Elman neural network improved by the genetic algorithm is used to obtain BPA, and the optimization method of GA is beneficial to improve the accuracy of BPA. The D-S evidence theory is improved, and the Pearson correlation similarity and evidence distance theory are used to revise the BPA, and then the evidence is fused according to the fusion rules, which effectively prevents the occurrence of inaccurate assessment decisions caused by evidence conflicts.
2. Propose a network security assessment model based on DS-GA-Elman. Through the analysis of the NSL-KDD dataset, the situation elements are extracted from the data according to the index system, the data are processed through quantitative formulas and normalization operations, and the dataset is divided, and the network security situation assessment model proposed in this paper is used to test the test. The final evaluation results have an accuracy rate of up to 80%.
3. Compared with the evaluation results of other models, the results of the network security situation evaluation model are better. In addition, this paper proposes a prediction model based on DEIPSO-GRU to predict the network security situation, and the predicted results are basically consistent with the actual situation value. The results are compared with the prediction results of other models, and it is verified that the network security situation prediction model proposed in this paper has a higher prediction accuracy.

## Figures and Tables

**Table 1:** Network security situation assessment indicator system.

First-level indicators	Secondary indicators
Availability	Traffic change rate, average survival time of major devices, mean time between failures of major devices, and total subnet traffic
Vulnerability	Total number of security devices, total number of open ports on major devices, number and extent of network vulnerabilities, device system type, and memory capacity
Threatening	Type and number of alarms, type and severity of attacks, historical frequency of attack events, and distribution and size of threat data packets
Disaster tolerance	Network topology, network bandwidth, number of concurrent threads of subnet servers, operating system types of subnet devices, and types of services provided

**Table 2:** Network security assessment level.

Security level	Description of network operation status
Class I (safety)	Network service is not affected
Class II (relatively safe)	Network service is slightly affected
Class III (basic safety)	Network services are moderately affected
Class IV (less safe)	Network services are severely affected
Class V (unsafe)	Network services are severely affected

**Table 3:** BPA value corresponding to the network security assessment level.

Security level	Situation BPA value
Class I (safety)	(0.8–1.0]
Class II (relatively safe)	(0.45–0.8]
Class III (basic safety)	(0–0.45]
Class IV (less safe)	(0.55–1.0]
Class V (unsafe)	(0–0.55]

**Table 4:** Evidence of cybersecurity posture based on BPA values.

Sample number	O_1_	O_2_	O_3_	U
4	0.023	0.098	0.821	0.031
15	0.241	0.398	0.162	0.148
26	0.346	0.443	0.119	0.144
42	0.279	0.398	0.251	0.039
65	0.109	0.406	0.239	0.261
74	0.152	0.374	0.279	0.163
81	0.056	0.178	0.706	0.037
98	0.197	0.218	0.499	0.042
88	0.118	0.235	0.507	0.139
93	0.326	0.102	0.428	0.118

**Table 5:** Evaluation results of D-S, Elman, GA-Elman, and DS-GA-Elman.

Samples	D-S	Elman	GA-Elman	DS-GA-Elman	Desired security level
4	VI	IV	IV	V	IV
15	III	III	III	III	III
26	III	II	III	III	III
42	III	II	II	II	II
65	III	II	III	III	III
74	IV	III	II	III	III
81	IV	IV	IV	IV	IV
98	V	V	IV	IV	V
88	V	IV	V	V	V
93	V	IV	V	IV	IV

**Table 6:** Test results of various evaluation algorithms.

Algorithm category	Correct number	Correct rate (%)
D-S	7	70
Elman	7	60
GA-Elman	6	70
DS-GA-Elman	8	80

**Table 7:** MAPE value corresponding to GRU, PSP-GRU, and DEIPSO-GRU algorithm.

Algorithm category	MAPE value	Prediction correctness
GRU	0.042	Lower
PSP-GRU	0.023	Medium
DEIPSO-GRU	0.014	Higher

## Data Availability

The dataset can be accessed upon request.
